# Gaviscon^® ^vs. omeprazole in symptomatic treatment of moderate gastroesophageal reflux. a direct comparative randomised trial

**DOI:** 10.1186/1471-230X-12-18

**Published:** 2012-02-23

**Authors:** Denis Pouchain, Marc-André Bigard, François Liard, Marc Childs, Annick Decaudin, Donna McVey

**Affiliations:** 1 Département de Médecine Générale, Université François Rabelais, Faculté de Médecine, 10, boulevard Tonnellé, BP 3223,6, rue du Docteur Lebel 94300, Vincennes 37032, Tours Cedex 1, France; 2 CHU de Nancy, Hôpital de Brabois, rue du Morvan, 54500 Vandœuvre-lès-Nancy, France; 3 General Practice, 37800 Saint-Epain, France; 4 Mediscan, 20, rue Saint-Saëns, 75015 Paris, France; 5 Reckitt Benckiser Healthcare France, 15, rue Ampère, 91748 Massy Cedex, France; 6 Reckitt Benckiser Group plc, 103-105 Bath Road, Slough, Berkshire SL1 3UH, UK

**Keywords:** Alginate, Gastroesophageal reflux disease (GERD), General practice, Omeprazole, Randomised controlled trial

## Abstract

**Background:**

Medical management of GERD mainly uses proton pump inhibitors. Alginates also have proven efficacy. The aim of this trial was to compare short-term efficacy of an alginate (Gaviscon^®^, 4 × 10 mL/day) and omeprazole (20 mg/day) on GERD symptoms in general practice.

**Methods:**

A 14-day multicentre randomised double-blind double-dummy non-inferiority trial compared Gaviscon^® ^(4 × 10 mL/day) and omeprazole (20 mg/day) in patients with 2-6 day heartburn episodes weekly without alarm signals. The primary outcome was the mean time to onset of the first 24-h heartburn-free period after initial dosing. Secondary outcomes were the proportion of patients without heartburn by D7, pain relief by D7, and reduction in pain intensity by D7 and D14.

**Results:**

278 patients were recruited; 120 were included in the Gaviscon^® ^group and 121 in the omeprazole group for the per protocol non-inferiority analysis. The mean time to onset of the first 24-h heartburn-free period after initial dosing was 2.0 (± 2.2) days for Gaviscon^® ^and 2.0 (± 2.3) days for omeprazole (*p *= 0.93); mean intergroup difference was 0.01 ± 1.55 days (95% CI = -0.41 to 0.43): i.e., less than the lower limit of the 95% CI of -0.5 days predetermined to demonstrate non-inferiority. The mean number of heartburn-free days by D7 was significantly greater in the omeprazole group: 3.7 ± 2.3 days vs. 3.1 ± 2.1 (*p *= 0.02). On D7, overall quality of pain relief was slightly in favour of omeprazole (*p *= 0.049). There was no significant difference in the reduction in pain intensity between groups by D7 (*p = *0.11) or D14 (*p = *0.08). Tolerance and safety were good and comparable in both groups.

**Conclusion:**

Gaviscon^® ^was non-inferior to omeprazole in achieving a 24-h heartburn-free period in moderate episodic heartburn, and is a relevant effective alternative treatment in moderate GERD in primary care.

**Trial registration:**

ISRCTN62203233.

## Background

In Western countries, 20% to 40% of adults suffer from episodes of heartburn due to gastroesophageal reflux disease (GERD) [1]. In France, a questionnaire study of 8,000 adults representative of the general population found a 31.3% prevalence of GERD symptoms. GERD was moderate (symptoms at least once a week) in 7.8% of cases (6% in under-50 year-olds, 10% in over-50s) [2]. Most (86%) moderate GERD sufferers had consulted for their symptoms, but 26% had delayed for more than one year, usually because they were not worried and/or were self-medicating. Treatment was monotherapy in two-thirds of cases: proton pump inhibitors (PPI) in 45% of cases, and antacids or alginates in 46%. Treatment was judged satisfactory by two-thirds of patients [2].

The efficacy of PPIs in symptomatic treatment of heartburn without esophagitis is well established [3,4]. The level of evidence is weaker for alginates (raft-forming oral suspensions/formulations), as the old comparative trials were on small samples: 286 patients overall in six trials vs. placebo [5-9]. Moreover, symptomatic efficacy is hard to assess for alginates, as formulae differ from country to country, with floating gel resistance varying by a factor of three [10,11].

In case of GERD symptoms without esophagitis on endoscopy or where endoscopy is not considered necessary (esophagitis prevalence in the general population not exceeding 2%) [1], treatment aims at rapid relief of symptoms (heartburn, acid regurgitation).

There have been no studies with a modern scientific double-blind, double-dummy design directly comparing one alginate to a PPI with heartburn as the primary clinical endpoint. We therefore performed a trial called "Gaviscon^® ^vs. Omeprazole in symptOmatic treatment of moDerate gastroesophageal reflux" (GOOD), the aim of which was to compare short-term symptomatic efficacy and safety between an alginate (Gaviscon^®^, 4 × 10 mL/day) and a PPI (omeprazole 20 mg/day) in moderate GERD in a general practice setting.

## Methods

### Design

The GOOD trial was a 14-day multicentre randomised double-blind double-dummy non-inferiority trial comparing efficacy between Gaviscon^® ^and omeprazole 20 mg. It recruited 90 general practitioners (GPs) so as to obtain 75 active investigators. Patients recorded symptoms 4 times a day for 2 weeks and the time of taking each treatment (morning, midday, evening and bedtime); they also recorded any onset of heartburn, and if so, at what time of day (morning, midday, evening, bedtime) and any experience of relief, and if so, at what interval after first taking the treatment. The GPs performed three mandatory assessments: D0 (inclusion visit), D7, and D14.

The trial ran from August 27 to November 29, 2010, and respected the ethical principles of the Seoul revision (2008) of the Helsinki Declaration and Good Clinical Practice. The study protocol received approval by the *Comité de Protection des Personnes d'Île-de-France VIII *ethics committee on May 3, 2010, and was registered (N° A 100 546-10) by the *Agence Française de Sécurité Sanitaire des Produits de Santé *(French health products approval authority). All patients were duly informed of the trial objectives and signed an informed consent form.

### Study population

Included patients were male or female, aged between 18 and 60 years, with 2 to 6 days of GERD episodes per week, with heartburn, with or without regurgitation, not taking alginate/antacid or PPI treatment for at least the preceding 2 months, and able to understand the study and to complete the self-administered questionnaires. Women of child-bearing age had to have effective birth control. Exclusion criteria were: atypical digestive or extradigestive symptoms without heartburn; gastric or duodenal ulcer; history of upper digestive tract surgery or of upper digestive tract or otorhinolaryngologic neoplasm; known hypersensitivity to at least one component of Gaviscon^® ^or of omeprazole; known hypersensitivity to benzimidazoles; and treatment with clopidogrel, atazanavir combined with ritonavir, ketoconazole, or itraconazole. Breastfeeding women and women who knew that they were pregnant as well as patients who had participated in a therapeutic trial within the month preceding inclusion in this trial were also excluded.

Included patients were randomly allocated to one of two groups: Gaviscon^® ^(4 × 10 mL/day), or omeprazole 20 mg/day. Randomisation by blocks of 3 (2 + 1) was double-blind. Successive blocks were balanced by 2 s.

### Study products

Gaviscon^® ^suspension in a 150-mL bottle (Reckitt Benckiser Healthcare France) was administered orally at a daily dose of 10 mL (2 teaspoonfuls), 4 times a day (after the three main meals and at bedtime). Omeprazole (omeprazole MYLAN^® ^20 mg, Mylan, France) in enteric coated capsule form was administered at a daily dose of 20 mg in the morning. Maximum treatment duration was 14 days.

The active substances of the Gaviscon^® ^oral suspension were sodium alginate and sodium bicarbonate. The placebo was composed of hydrogenated glucose syrup, xanthane gum, methyl parahydroxybenzoate (E218), propyl parahydroxybenzoate (E216), erythrosine (E217), fennel flavour, titanium oxide, and purified water. The placebo was developed so as to have the same aspect, colour, odour and flavour as the aniseed Gaviscon^® ^suspension.

All Gaviscon^® ^group patients also received a capsule of omeprazole-placebo every morning for 14 days, and all omeprazole 20 mg group patients also received 10 mL of Gaviscon^®^-placebo 4 times a day (after the three main meals and at bedtime) for 14 days.

Study drugs were packaged per patient and per site according to the randomisation list.

### Endpoints

The primary outcome was the mean time to onset of the first 24-h heartburn-free period after initial dosing. This outcome was assessed by the GP, based on the self-administered questionnaire filled in 4 times a day by the patient. Mean time to onset was calculated as the difference between 2 time-points: the time of taking the treatment for the first time and the date and time (morning, midday, evening, before bedtime) at which a 24-h heartburn-free period had been achieved.

Secondary outcomes were: (a) the mean number of days without heartburn by D7 as assessed from the patient's self-administered questionnaire; (b) patient's overall qualitative self-assessment of pain relief on D7 on a 5-point Likert scale; and (c) pain intensity on D7 and D14, assessed by the patient on a 100-mm visual analog scale (VAS).

### Adverse events

Adverse events (AEs) were collected at the two study visits (D7 and D14). An AE was defined as an untoward medical event that occurred during the study period, whether or not related to the study procedure or study products.

Severe AE (SAE) was defined as an untoward medical event that resulted in death, was life-threatening, required inpatient admission or prolongation of hospitalization, or resulted in severe or persistent disability or incapacity.

### Statistical analysis

Descriptive statistical analyses were performed on the data collected on D0, D7 and D14, and those of the self-administered questionnaire (D0 to D7, and D7 to D14), for the intention-to-treat (ITT) and per protocol (PP) populations. The PP population included all patients from the ITT population who attended at least one of the study visits, except those with major protocol deviations liable to interfere with the primary outcome result. As the objective of the GOOD trial was to determine whether Gaviscon^® ^was non-inferior to omeprazole 20 mg, the PP population was the reference for efficacy analysis, and the efficacy results presented here are those for the PP population.

Inter-group statistical comparison used appropriate one-tailed tests: Chi^2 ^test for qualitative variable (or Fisher's exact test in case of sample size < 5), Student *t*-test for Gaussian quantitative variables, and non-parametric Wilcoxon test for semi-quantitative or non-Gaussian quantitative variables.

Group comparison used analysis of variance (ANOVA) when variable distribution was normal. Inter-group comparability was checked at inclusion for heartburn frequency and severity, GERD duration, age, regurgitation and alcohol consumption. In case of non-comparability, analysis of covariance (ANCOVA) was performed, introducing into the model the variable or variables that were non-comparable at baseline.

If the distribution of time to a 24-h heartburn-free period was non-normal, a non-parametric Wilcoxon test was used, with the Hodges-Lehmann median estimated with its 95% confidence interval (CI).

Statistical analysis was carried out on SAS version 8.2 (SAS Institute, North Carolina, USA). The significance threshold was set at 5%.

### Choice of the lower limit

In non-inferiority studies, the lower limit is classically set at 50% of the reference substance effect in comparison to placebo [12]. Previous studies showed that the time to a 24-h heartburn-free period was 19-21 days with placebo [13], 4-5 days with rabeprazole 20 mg [13], and 2 days with pantoprazole 20 mg or omeprazole 20 mg [14] (at least 16 days' difference between placebo and PPI). Halving this difference gives a non-inferiority limit of 8 days, which was neither ethically nor clinically acceptable.

The most recent study [14] reported a value of 1.8 ± 0.8 day for omeprazole 20 mg. The GOOD trial hypothesized that omeprazole 20 mg provides a time to onset of the first 24-h heartburn-free period of 2 ± 1 days. As patients recorded symptoms 4 times a day, the non-inferiority of Gaviscon^® ^would be demonstrated by a clinically relevant value of 0.5 days less than for omeprazole 20 mg.

For the non-inferiority test, the mean time difference in onset of the first 24-h heartburn-free period between treatment groups was used, with its 95%CI. If the lower limit (0.5 days) was within this confidence interval, the non-inferiority hypothesis was taken to be confirmed.

### Sample size calculation

For an α-risk of < 5% and power of 95%, the requisite sample size was 88 assessable patient data sets per group. To allow for incomplete recording of symptoms by patients and loss to follow-up (< 10%), 30% extra patients were to be recruited: i.e., 120 patients per group. In all, 240 patients were required in order to meet the primary endpoint. 90 investigation centres were set up, recruiting three patients each with a 3.5 month deadline.

## Results

**Figure 1:**
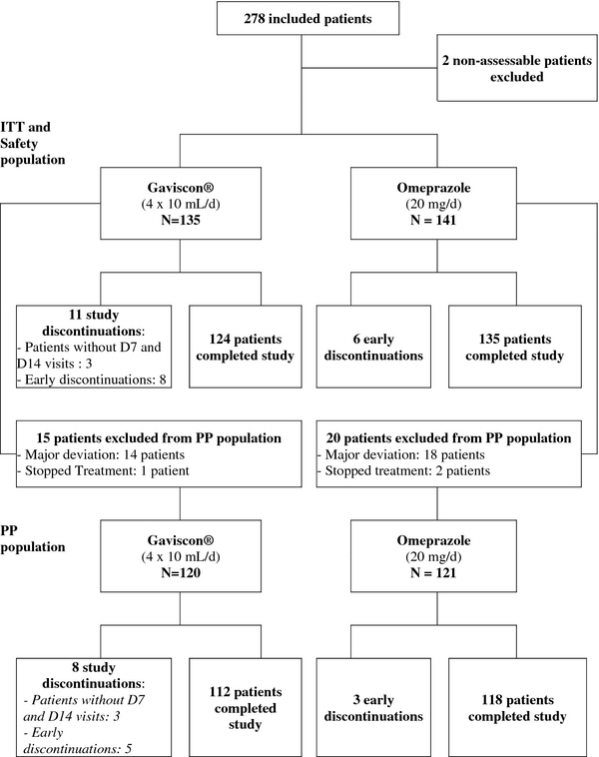
**Study patients flowchart**.

278 patients were recruited by 75 French GPs and 241 included for efficacy analysis in the PP population: 120 in the Gaviscon^® ^group and 121 in the omeprazole 20 mg group (Figure 1).

At inclusion, PP population characteristics were comparable between groups (Table 1) and did not differ from those of the ITT population (data not shown).

**Table 1:** Baseline characteristics of the patients (per protocol population)

	Gaviscon^®^ N = 120	Omeprazole N = 121
Age, years: mean (SD)	46.3 (10.7)	44.5 (12.2)

Women: N (%)	73 (60.8)	65 (53.7)

Weight, kg: mean (SD)	73.3 (14.8)	74.5 (16.1)

BMI, kg/m^2^: mean (SD)	26.0 (5.2)	26.2 (4.7)

BMI ≥ 30 kg/m^2^: N (%)	19 (15.8)	23 (19.0)

GERD, time to diagnosis, years: mean (SD)	4.7 (6.3)	5.3 (7.3)

History of upper endoscopy: N (%)	34 (28.3)	44 (36.4)

Number of days with heartburn per week		

- 2: N (%)	9 (7.5)	6 (5.0)

- 3: N (%)	29 (24.2)	24 (19.8)

- 4: N (%)	35 (29.2)	27 (22.3)

- 5: N (%)	17 (14.2)	28 (23.1)

- 6: N (%)	30 (25.0)	36 (29.8)

- Mean: N (SD)	4.3 (1.3)	4.5 (1.2)

Heartburn		

- Mild: N (%)	10 (8.3)	15 (12.4)

- Moderate: N (%)	83 (69.2)	73 (60.3)

- Severe: N (%)	27 (22.5)	33 (27.3)

Heartburn-related pain (VAS), mm: mean (SD)	52 (22)	53 (22)

Systolic BP, mmHg: mean (SD)	124.8 (10.8)	125.4 (11.0)

Diastolic BP, mmHg: mean (SD)	74.4 (8.5)	74.2 (8.2)

Heart rate, beat/min: mean (SD)	72.6 (7.5)	72.1 (9.0)

Smoking, Yes: N (%)	26 (21.7)	27 (22.3)

Alcohol consumption, Yes: N (%)	29 (24.2)	37 (30.6)

Living alone: N (%)	25 (20.8)	37 (30.6)

In couple: N (%)	95 (79.2)	84 (69.4)

Place of residence		

- Rural	37 (30.8)	37 (30.6)

- Suburban	35 (29.2)	34 (28.1)

- Town	33 (27.5)	25 (20.7)

- City	15 (12.5)	25 (20.7)

Occupationally active	91 (75.8)	80 (66.1)

The mean age of included patients (PP population) was 45.4 ± 11.5 years (SD). Mean body mass index (BMI) was 26.1 ± 5.0 kg/m^2^, and 20% of patients had BMI ≥ 30 kg/m^2^. Patients had suffered from GERD for a mean 6.5 ± 7.1 years and reported a mean 4.4 days of heartburn episodes in the week preceding inclusion.

Before inclusion, 78 patients (32.4%) underwent endoscopy: results were normal for 34.6%; 30.8% showed hiatus hernia and 28.2% mild to moderate esophagitis. No patients reported history of severe esophagitis. Alcohol consumption and smoking were moderate and comparable between groups.

The percentage of patients with very good or good compliance with medication was similar (*p *= 0.08) between the Gaviscon^® ^(95.7%) and omeprazole (95.9%) groups.

### Efficacy

#### Primary outcome

Mean time to onset of the first 24-h heartburn-free period after initial dosing was 2.0 ± 2.2 days in the Gaviscon^® ^group vs. 2.0 ± 2.3 days in the omeprazole group (*p *= 0.93). Mean intergroup difference was 0.01 ± 1.55 days (95% CI:-0.41 to 0.43): i.e., less than the lower limit of the predetermined 95% CI (-0.5), thus demonstrating the non-inferiority of Gaviscon^® ^compared to omeprazole 20 mg (Table 2).

**Table 2:** Mean time to onset of the first 24-h heartburn-free period after initial dosing and number of patients free from heartburn for 24 h (per protocol population)

	Gaviscon^®^ N = 120	Omeprazole N = 121	*p-*value
Mean time to the onset of a 24-h heartburn-free period, days: mean (SD)	2.0 (2.2)	2.0 (2.3)	0.93

Median, days	1.2	1.2	

Number of patients free of heartburn for 24 h: N (%)	105 (89.7)	109 (90.1)	0.93

The proportion of patients achieving a first 24-h heartburn-free period was 105/120 (89.7%) in the Gaviscon^® ^group, vs. 109/121 (90.1%) in the omeprazole group (*p *= 0.93).

#### Secondary outcomes

The mean number of heartburn-free days by D7 was significantly greater in the omeprazole 20 mg than in the Gaviscon^® ^group: 3.7 ± 2.3 vs. 3.1 ± 2.1 days (absolute difference = 0.6 days; *p *= 0.02) (Table 3).

**Table 3:** Mean number of days without heartburn by D7 (per protocol population)

		Gaviscon^®^ N = 120 (%)	Omeprazole N = 121(%)	*p-*value
Number of days without heartburn	0	19 (16.5)	15 (12.7)	0.33
		
	1	10 (8.7)	10 (8.5)	
		
	2	17 (14.8)	12 (10.2)	
		
	3	22 (19.1)	16 (13.6)	
		
	4	18 (15.7)	16 (13.6)	
		
	5	10 (8.7)	15 (12.7)	
		
	6	13 (11.3)	20 (16.9)	
		
	7	6 (5.2)	14 (11.9)	
	
	Mean (SD)	3.1 (2.1)	3.7 (2.3)	0.02

By D7, overall self-assessed qualitative pain relief was also in favour of omeprazole: *p *= 0.049 (Table 4).

**Table 4:** Overall quality of pain relief by D7 (per protocol population)

	Gaviscon^®^ N = 120 (%)	Omeprazole N = 121 (%)	*p-*value
Much worse	0 (0)	1 (0.8)	0.049
	
A little worse	4 (3.4)	1 (0.8)	
	
No change	10 (8.5)	5 (4.2)	
	
A little better	41 (35.0)	38 (31.9)	
	
Much better	62 (53.0)	74 (62.2)	

There was no significant difference between groups in clinically relevant reduction in pain intensity: *p *= 0.11 by D7 and *p *= 0.08 by D14 (Table 5).

**Table 5:** Pain intensity by D0, D7 and D14: 100-mm visual analog scale (per protocol population)

	Gaviscon^®^ N = 116	Omeprazole N = 118	*p-*value
D0, mm, mean (SD)	51 (22)	53 (22)	0.52

D7, mm, mean (SD)	17 (17)	14 (17)	0.11

Δ D0-D7, mm, mean (SD)	-34 (27)	-39 (26)	0.12

Day 14, mm, mean (SD)	13 (18)	10 (16)	0.08

Δ D0-D14, mm, mean (SD)	-38 (27)	-44 (27)	0.11

#### Safety

All patients took at least one dose of Gaviscon^® ^or omeprazole 20 mg, and were thus included in the safety analysis. Results are therefore presented for the ITT population.

Seventeen (12.6%) of the 135 patients in the Gaviscon^® ^group vs. 20 (14.2%) of the 141 patients in the omeprazole 20 mg group experienced at least one AE during the study (*p *= 0.70). The percentage of patients with at least one AE was also comparable, regardless of time period: 9.1% in the Gaviscon^® ^group vs. 9.2% in the omeprazole group between D0 and D7 (*p *= 0.97) and respectively 5.5% vs. 5.8% between D7 and D14 (*p *= 0.91).

The most frequently observed AEs were nausea (1.8%), constipation (1.5%), rhinopharyngitis (1.5%), drug intolerance (1.1%), abdominal pain, diarrhoea, abdominal distension, rhinitis and cough (0.7% each). All other AEs had an incidence of 0.4% each.

One patient in the omeprazole 20 mg group experienced one SAE (bowel obstruction).

## Discussion

The GOOD trial is the first randomised controlled double-blind, double-dummy trial to directly compare efficacy between an alginate and a PPI with heartburn as the clinical primary endpoint. It showed that Gaviscon^® ^(4 × 10 mL/day) was not inferior to omeprazole 20 mg/day in achieving onset of the first 24-h heartburn-free period after initial dosing in patients with moderate GERD (heartburn at least once a week). Mean times to onset of the first 24-h heartburn-free period after initial dosing were 2 ± 2.2 days for Gaviscon^® ^and 2 ± 2.3 days for omeprazole (*p *= 0.93). In both groups, 9 out of 10 patients had a heartburn-free period of at least 24 h.

The mean number of heartburn-free days by D7 was significantly (*p *= 0.02) greater with omeprazole, due to a higher rate of symptom recurrence in the Gaviscon^® ^group. The weekly absolute difference was 0.6 days.

There was no significant difference between groups in clinically relevant reduction in pain intensity, although overall qualitative pain relief was slightly (*p *= 0.049) in favour of omeprazole.

Results of the GOOD trial also showed that Gaviscon^® ^and omeprazole 20 mg could be used both safely.

This trial, performed in a general practice setting, included patients with symptoms highly suggestive of GERD (heartburn, regurgitation) and with a very low estimated risk of ulcerative esophagitis. The same primary outcome was used in a trial comparing rabeprazole vs. placebo in GERD patients without erosive or ulcerated esophagitis on endoscopy [13]. In the rabeprazole 20 mg/day group, the median time to a 24-h heartburn-free period was 4.5 days, vs. 21.5 with placebo. A trial comparing pantoprazole 20 mg/day and esomeprazole 20 mg/day in GERD without esophagitis found a median 2 days to the beginning of heartburn relief in both groups [14].

Alginates showed proven efficacy against GERD symptoms in randomised trials vs. placebo [5-9]. As the medications in both arms of the GOOD trial had proven short-term symptomatic efficacy in GERD vs. placebo, no placebo arm was deemed necessary.

The limitation of the GOOD trial was the treatment period, which was only 14 days, with the primary outcome set during the first 7 days of treatment. The objective was to determine whether Gaviscon^® ^could be a relevant alternative to omeprazole 20 mg in patients suffering from mild-to-moderate episodic GERD in primary care, not requiring prolonged continuous treatment. These patients represent 74% of those consulting a primary care physician with a GERD complaint [2], and require short-term treatment only. As the primary outcome was the time to onset of the first 24-h heartburn-free period, each day was divided into four periods so as to have four symptom assessments per day. As the treatment was symptomatic, this endpoint was relevant clinically and from the patient's point of view. Patients in the GOOD trial had moderate GERD, and therefore probably belonged to the population of patients able to use over-the-counter PPIs, which in France are packaged for a 14-day course of treatment.

The comparison was between two drugs with different pharmacokinetic and pharmacodynamic properties. Omeprazole 20 mg is somewhat pharmacologically effective as of D1, inhibiting 70% of proton pumps, with efficacy increasing up to D3 [15]. Alginates display immediate action, forming a raft floating over the stomach contents, eliminating or displacing the postprandial "acid pocket", so that, in case of reflux, the raft is regurgitated first into the lower oesophagus, reducing acid contact, especially when the subject is standing [16-21]. The raft may remain in the stomach for several hours [18] but is then evacuated, so that 3 or 4 doses per day are required for optimal efficacy.

The trial used a simple, relevant and pragmatic primary clinical endpoint rather than a composite score such as symptom frequency plus intensity. On the secondary endpoints, the results were marginally (*P *= 0.049) in favour of better efficacy for omeprazole 20 mg on overall qualitatively perceived pain relief (but not on pain intensity) and mean number of heartburn-free days between D0 and D7, were in agreement with many literature reports of omeprazole 20 mg's efficacy on reflux symptoms with or without esophagitis [20].

In the last 15 years, several guidelines have been published [22-28], but mainly focused on GERD requiring a medical opinion from a gastroenterologist (disabling and/or continuous symptoms, esophagitis on endoscopy, extra-digestive manifestations, and treatment failure). In these patients, alginates and antacids were often restricted to self-medication. The GOOD trial demonstrated a role for Gaviscon^® ^in the management of moderate GERD with occasional recurrence, alongside on-demand PPIs in patients showing rapid response to the latter [29]. This is a relevant and useful alternative and an effective non-systemic approach that should help reduce excessive use of curative or preventive prescriptions of PPIs [30]. PPIs are a well-tolerated pharmacologic class, but concomitant prescription of omeprazole with clopidogrel should be managed carefully after coronary stenting [31-33]. Some authors suggested that prolonged PPI therapy could increase *Clostridium difficile *infection [34], community-acquired pneumopathy [35] and risk of hip fracture [36,37], so that this pharmacologic class should be prescribed in moderation if other safe rapid-relief solutions are available.

## Conclusion

In a general practice setting for patients complaining of moderate heartburn, Gaviscon^® ^(4 × 10 mL/day) is an effective short-term treatment option in mild-to-moderate GERD, in terms of onset of a first 24-h heartburn-free period after initial dosing. It proved non-inferior to omeprazole 20 mg/day, and is thus a relevant and effective alternative treatment in case of moderate and episodic symptoms of GERD as managed in general practice.

## Competing interests

Denis Pouchain, MD: served as a consultant for Reckitt Benckiser Healthcare France and Bayer Healthcare Consumer Care and received funding from Reckitt Benckiser Healthcare France to lead this trial.

Marc-André Bigard, MD: served as speaker, consultant and advisory board member for Abbott, AstraZeneca, Bayer Healthcare, Ferring, IPRAD, Sanofi, Schering-Plough, and Reckitt Benckiser Healthcare France.

François Liard, MD: served as speaker, consultant and advisory board member for Lündbeck, Reckitt Benckiser Healthcare France, Bayer Healthcare, Menarini, Sanofi, Pfizer, and Biocodex.

Marc Childs, MD: employee of Mediscan, CRO in charge of the study funded by Reckitt Benckiser Healthcare France.

Annick Decaudin, PharmD: Reckitt Benckiser Healthcare, France.

Donna McVey, MD: Reckitt Benckiser, United Kingdom.

## Authors' contributions

DP, MAB and MC designed the study. FL participated in the design. MC conducted the data collection and designed and carried out the statistical analysis. DP wrote the article. All authors participated in critical revision of the manuscript and have seen and approved the final version. DP is the guarantor of this trial.

## Pre-publication history

The pre-publication history for this paper can be accessed here:

http://www.biomedcentral.com/1471-230X/12/18/prepub
